# Bioorganometallic tagging of N-acetylhistamine with an Fe(CO)_3_ unit: synthesis, X-ray structure, and protonation behavior

**DOI:** 10.55730/1300-0527.3773

**Published:** 2025-10-28

**Authors:** Salah MERNIZ, Louiza HIMED, Rofia DJERRI, Belkis AKACHAT

**Affiliations:** 1 Institute of Industrial Hygiene and Safety, University of Batna 2, Batna, Algeria; 2 Division of Biotechnology and Food Quality (BIOQUAL-INATAA), Department of Food Biotechnology, Institute of Nutrition, Food and Agro-Food Technologies, University of Freres Mentouri Constantine 1, Constantine, Algeria

**Keywords:** Iron carbonyl complexes, molecular labeling, acid-base analysis, Fourier transform infrared (FTIR) spectroscopy, single-crystal X-ray diffraction study

## Abstract

This study highlights the potential of organometallic carbonyl complexes as selective markers for biomolecules, enabling sensitive infrared (IR) detection. The regio- and stereoselective coupling of N-acetylhistamine, a histidine analogue, with the precursor complex 1 [Fe(CO)_3_(1,4-η^5^-N-pyridiniocyclohexa-1,3-diene)] BF_4_ affords the labeled complex 3 [Fe(CO)_3_(1,4-η^5^-N-acetylhistaminocyclohexa-1,3-diene)]. X-ray diffraction (XRD) confirms the exo stereochemistry and reveals a rigid, well-defined architecture. IR and ^1^H nuclear magnetic resonance spectroscopic studies combined with IR-monitored acid–base titration demonstrate the complex’s stability in aqueous media between pH 5 and 8, alongside a modest increase in basicity relative to the free ligand. These findings establish the Fe(CO)_3_ moiety as a robust platform for selective labeling of peptides and proteins, paving the way for targeted applications in bioorganometallic chemistry and spectroscopic imaging.

## 1. Introduction

The selective covalent incorporation of transition-metal complexes into biomolecules has emerged as a powerful strategy for probing and manipulating biological systems at the molecular level [1,2]. Among these, metal carbonyl complexes are distinguished by their unique infrared (IR) signatures, characterized by intense and well-defined C≡O stretching bands, which make them exceptional spectroscopic probes for elucidating biomolecular microenvironments [3].

**Figure 1:**
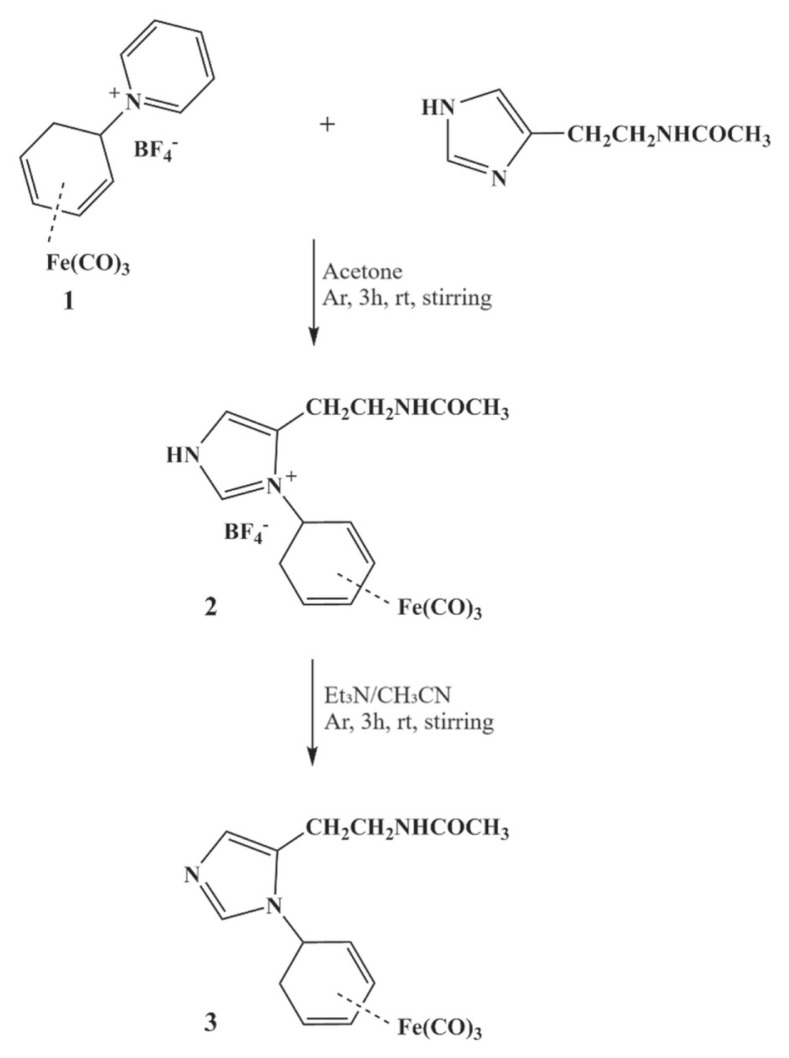
Schematic representation of the coupling process between N-acetylhistamine and the labeling complex 1.

Pioneering work by Jaouen and coworkers demonstrated the covalent tagging of estradiol with bimetallic molybdenum complexes exhibiting high affinity for the cytosolic estradiol receptor isolated from sheep uterus. The resulting covalent linkage can be readily monitored by Fourier transform infrared (FTIR) spectroscopy through characteristic carbonyl vibrations, providing direct insight into ligand–receptor interactions [4,5]. This methodology has since been extended to other complexes, notably Cr(CO)_3_-based systems, confirming the robustness and generality of this approach [6]. Beyond conventional spectroscopy, this concept has inspired the development of innovative immunoassays leveraging the IR properties of carbonyl complexes, giving rise to the so-called “carbonyl metallo immuno assay.” This technique offers a nonradioactive, highly sensitive alternative for trace biomarker quantification by exploiting the spectroscopic detection of carbonyl signals [7]. The versatility of these organometallic probes largely relies on synthetic strategies enabling their covalent introduction into complex macromolecules. In particular, the regio- and stereoselective addition of N- and S-nucleophiles to the cationic intermediate [Fe(CO)_3_(1,5-η^5^-C_6_H_7_)]^+^ affords exclusively exoconfigured C_5_–Y (Y = N, S) bonds, ensuring remarkable complex stability in aqueous media and compatibility with delicate biological systems [8]. Recent advances in molecular imaging have further expanded the applications of these carbonyl complexes to subcellular visualization. Infrared photothermal induced resonance spectroscopy now enables nanoscale mapping of metal carbonyl complexes within living cells without fluorescent labeling and offers unprecedented spatial resolution [9]. Such developments open new avenues for in situ studies of metallobiomolecular interactions in complex environments. Herein, we report the synthesis, reactivity, and single-crystal X-ray diffraction (SCXRD) structural characterization of a novel complex, 3 [Fe(CO)_3_(1,4-η^5^-N-acetylhistaminocyclohexa-1,3-diene)] (Figure 1). This complex was obtained via covalent coupling between the cationic [Fe(CO)_3_(1,4-η^5^-N-pyridiniocyclohexa-1,3-diene)] BF_4_ 1 and the biologically relevant N-acetyl histamine, an analogue of histidine. N-acetyl histamine was selected as a model amine-bearing substrate due to its dual relevance: it features a primary amine group suitable for nucleophilic substitution, and it is a biologically active derivative of histamine. This choice provides a representative system for exploring the covalent tagging of bioactive amines using organometallic Fe(CO)_3_ fragments.

We used FTIR spectroscopy to monitor the acid–base behavior of the labeled complex 3, bearing an N-acetyl histamine ligand (HNhist) sensitive to solution pH.

## 2. Experimental section

### 2.1. Instrumentation and analytical methods

IR spectra were recorded on a Bruker VERTEX 70v FTIR spectrometer equipped with an attenuated total reflectance accessory, ensuring high sensitivity and reproducibility. Spectra were collected over 4000–400 cm^−1^ with a resolution of 4 cm^−1^. When needed, KBr pellet preparations were also used. Absorption bands are reported in wavenumbers (cm^−1^). ^1^H Nuclear Magnetic Resonance (^1^H NMR) spectra were recorded on a Bruker Avance III 400 MHz spectrometer at room temperature. Chemical shifts (δ) are reported in ppm relative to tetramethylsilane (TMS) as the internal standard. Coupling constants (*J*) are reported in hertz (Hz). SCXRD data were collected on a Bruker D8 Venture X-ray diffractometer equipped with a Photon 100 CMOS area detector, using monochromated Mo Kα radiation (λ = 0.71073 Å). Data collection, cell refinement, and data reduction were performed using the APEX4 Software package [10]. The structure of complex 3 was solved by direct methods with SHELXT 2018/2 [11] and refined on F^2^ by full-matrix least-squares techniques using SHELXL-2018/3 [12].

All hydrogen atoms, including the amide hydrogen atom (H3), were placed in geometrically calculated positions and refined using standard riding models with isotropic displacement parameters. Nonhydrogen atoms were refined anisotropically. Structural illustrations and packing diagrams were generated using ORTEP-3 [13] and Mercury 4.3.1 [14].

Crystallographic data for compound 3 have been deposited at the Cambridge Crystallographic Data Centre (CCDC) under deposition number 2421142 (see Supplementary information).

### 2.2. Synthetic procedures

All reactions were carried out under an inert argon atmosphere using standard Schlenk line techniques. Anhydrous solvents were rigorously dried and freshly distilled prior to use according to established procedures. The progress of the reactions was monitored by thin-layer chromatography and IR spectroscopy. Products were purified by flash column chromatography on silica gel, followed, when necessary, by recrystallization from appropriate solvents to ensure high purity and crystallinity. Final compounds were fully characterized by spectroscopic (IR, NMR) and analytical methods, providing unambiguous structural confirmation.

#### 2.2.1. Synthesis of complex 1 [Fe(CO)_3_(1,4-η^5^-N-pyridiniocyclohexa-1,3-diene)] BF_4_

Complex 1, [C_11_H_9_NFe(CO)_3_][BF_4_], was synthesized following a modified literature procedure for pyridinio-cyclohexadienyl iron tricarbonyl derivatives [15–17]. The compound was obtained in 70% yield after column chromatography and recrystallization.

IR (KBr, cm^−1^): ν(C≡O) = 2056, 1979; ν(B–F) = 1118, 1084, 1030.

^1^H NMR (250 MHz, CD_3_COCD_3_, δ in ppm vs. TMS): 2.00 (m, 1H), 2.90 (m, 1H), 3.35 (m, 2H), 5.55 (m, 1H), 6.00 (m, 2H), 8.18 (t, *J* = 7.7 Hz, 2H), 8.62 (t, *J* = 7.8 Hz, 1H), 9.10 (d, *J* = 5.6 Hz, 2H).

#### 2.2.2. Synthesis of complex 2 [Fe(CO)_3_(1,4-η^5^-N-acetylhistaminocyclohexa-1,3-diene)] BF_4_

Complex 2 was prepared by reacting complex 1 ([C_11_H_9_NFe(CO)_3_][BF_4_], 0.77 g, 2.0 mmol) with N-acetylhistamine (0.306 g, 2.0 mmol) in acetone (20 mL) under an inert argon atmosphere at room temperature. After 3 h of stirring, the reaction mixture was filtered and evaporated under reduced pressure. The residue was dissolved in CH_2_Cl_2_, washed with water, dried over MgSO_4_, and purified by flash chromatography on silica gel (diethyl ether/petroleum ether, 15:85 v/v), affording a pale yellow powder—yield: 75% (0.688 g).

IR (KBr, cm^−1^): ν(N–H) = 3419, 3260; ν(C–H) = 3019, 2855; ν(C≡O) = 2053, 1982; ν (amide I) = 1642; ν(amide II) = 1541; ν(B–F) = 1124, 1084, 1038.

^1^H NMR (250 MHz, CD_3_COCD_3_, δ in ppm vs. TMS): 1.87 (m, 4H, CH_3_ and H6), 2.73 (ddd, 1H, *J* = 15.6, 11.1, 3.7 Hz, H6’), 3.01 (t, 2H, *J* = 6.3 Hz, CH_2_–imidazole), 3.12 (t, 1H, *J* = 4.6 Hz, H1), 3.22 (m, 1H, H4), 3.46 (m, 2H, CH_2_–NH), 5.19 (dt, 1H, *J* = 11.2, 2.9 Hz, H5’), 5.87 (t, 1H, *J* = 5.2 Hz, H3), 5.96 (t, 1H, *J* = 4.8 Hz, H2), 7.54 (s, 1H, imidazole Hb), 8.99 (s, 1H, imidazole Ha).

The elemental composition of complex 2 was confirmed by carbon–hydrogen–nitrogen (CHN) elemental analysis. The experimentally determined values (C 40.96%, H 4.15%, N 8.91%) are consistent with the theoretical values calculated for C_16_H_18_BF_4_FeN_3_O_4_ (C 41.86%, H 3.92%, N 9.16%), supporting the proposed molecular formula and confirming the compound’s homogeneity and purity.

Complex 2 was isolated as a tetrafluoroborate salt, resulting from the reaction of the BF_4_-containing cationic precursor 1 with N-acetylhistamine. The formation of the protonated ammonium (histaminio) species occurs spontaneously during the substitution process, driven by internal proton transfer and the electron-deficient nature of the organometallic fragment. This salt form is supported by the ^1^H NMR spectrum, where the chemical shifts of the NH and imidazole protons indicate protonation, and confirmed by the elemental analysis.

No external acid was added during the synthesis. The salt form of complex 2 results from an internal proton transfer, allowing the reaction to proceed under mild, near-neutral, and aprotic conditions that are potentially compatible with sensitive biomolecular systems.

#### 2.2.3. Synthesis of complex 3 [Fe(CO)_3_(1,4-η^5^-N-acetylhistaminocyclohexa-1,3-diene)]

Complex 3 was obtained by treating a solution of the tetrafluoroborate precursor 2 (0.918 g, 2.0 mmol) in acetonitrile (20 mL) with equimolar triethylamine (0.278 mL, 2.0 mmol) under an inert atmosphere. After stirring for 30 min at ambient temperature, the reaction mixture was filtered and concentrated under reduced pressure. The resulting solid was triturated with toluene to efficiently remove pyridine byproducts, then dried under vacuum. Recrystallization from an acetone/pentane mixture furnished complex 3 as a pale yellow powder in 41% yield (0.302 g).

IR (KBr, cm^−1^): ν(N–H) = 3427, 3255; ν(C–H) = 3054, 2927, 2854; ν(C≡O) = 2047, 1980, 1943; ν(amide I) = 1672; ν(amide II) = 1566.

^1^H NMR (250 MHz, CD_3_COCD_3_, δ ppm vs. TMS): 1.71 (dt, 1H, *J* = 11.0, 3.0 Hz, H6), 1.83 (s, 3H, CH_3_ amide), 2.57 (m, 3H, H6’ and βCH_2_), 3.14 (t, 1H, *J* = 4.8 Hz, H1), 3.24 (t, 1H, *J* = 4.8 Hz, H4), 3.36 (m, 2H, CH_2_–NH), 4.80 (dt, 1H, *J* = 10.9, 3.0 Hz, H5’), 5.76 (t, 1H, *J* = 4.6 Hz, H3), 5.90 (t, 1H, *J* = 4.6 Hz, H2), 6.85 (s, 1H, Hb), 7.50 (s, 1H, Ha).

CHN elemental analysis was also conducted for complex 3 to assess its bulk purity. The observed values (C 51.60%, H 4.86%, N 11.14%) are in close agreement with the calculated values for C_16_H_17_FeN_3_O_4_ (C 51.78%, H 4.58%, N 11.32%). These data confirm the high purity and molecular integrity of the neutral complex.

This transformation highlights a straightforward and efficient protocol for accessing the neutral iron tricarbonyl complex bearing an N-acetylhistaminio ligand, with facile removal of byproducts and a moderate isolated yield.

### 2.3. Acid–base titration of complex 3 [Fe(CO)_3_(1,4-η^5^-N-acetylhistaminocyclohexa-1,3-diene)]

The acid–base behavior of complex 3 was investigated using FTIR spectroscopy. Aqueous solutions of the complex (5 μL, 0.001 M) were prepared and deposited onto 3 mm KBr pellets, then air-dried prior to analysis. The titrations were carried out in phosphate buffer (0.2 M) at pH 5.3, 7.0, 7.6, and 7.9, and in carbonate buffer (0.1 M) at pH 8.3, 9.0, and 10.1. FTIR spectra were recorded in the range 1800–2100 cm^−1^ to monitor the carbonyl stretching vibrations as a function of pH. These experiments enabled the assessment of the pH-dependent protonation–deprotonation behavior of the N-acetylhistamine ligand coordinated to the Fe(CO)_3_ unit.

## 3. Results and discussion

The targeted labeling of N-acetylhistamine was successfully achieved through regio- and stereoselective coupling with the organometallic precursor [Fe(CO)_3_(η^5^-C_6_H_7_N^+^)]BF_4_ 1, a well-known source of the electrophilic [Fe(CO)_3_(η^5^-C_6_H_7_)]^+^ moiety. This reaction proceeds cleanly to furnish the cationic complex [Fe(CO)_3_(η^5^-C_6_H_7_-N-acetylhistaminio)]BF_4_ 2 in 75% isolated yield. Notably, the transformation highlights the remarkable compatibility of the η^5^-pyridinium platform with nucleophilic biorelevant amines, enabling a straightforward entry to labeled histaminic derivatives. Subsequent treatment of complex 2 with triethylamine induces selective deprotonation of the histaminio moiety, affording the neutral complex [Fe(CO)_3_(η^5^-C_6_H_7_-N-acetylhistaminio)] 3 in 41% yield. This step further demonstrates the modular reactivity of the iron tricarbonyl framework and its suitability for tuning electronic and structural properties via simple acid–base manipulation (Figure 1).

### 3.1. Spectroscopic studies

#### 3.1.1. Infrared spectroscopy

The IR spectra of complexes 2 and 3, recorded as KBr pellets, exhibit two to three strong absorption bands in the 1900–2100 cm^−1^ region, characteristic of terminal carbonyl stretching vibrations (νC≡O) in iron tricarbonyl complexes. These bands are highly sensitive to the electron density at the metal center and the nature of the coordinated ligands [18–19].

Complex 3 displays a noticeable shift of the ν(CO) bands to lower wavenumbers compared to complex 2, indicating a stronger electron-donating effect from the N-acetylhistamine ligand relative to pyridine. The presence of multiple absorptions around 1100 cm^−1^ in the spectrum of 2 is consistent with a salt form, whereas 3 appears to be neutral [20]. Relative to the starting material 1, the ν(CO) bands of 3 are red-shifted by 3 to 9 cm^−1^. This shift is attributed to enhanced metal-to-CO back-donation, resulting from increased σ-donor strength of the organic ligand [21–22]. The stronger electron donation from N-acetylhistamine increases the electron density on the Fe center, thereby reinforcing backbonding into the π* orbitals of the carbonyl ligands and weakening the C≡O bond, which lowers the vibrational frequency. Recent studies have confirmed that such IR shifts serve as sensitive probes for electronic interactions in Fe–CO systems, especially when biologically relevant N-donor ligands are involved [23]. Moreover, DFT-assisted IR analysis provides reliable insight into the electronic structure and ligand effects in carbonyl complexes [24].

#### 3.1.2. ^1^H NMR spectroscopy

The ^1^H NMR spectra of complexes 2 (protonated) and 3 (neutral) display diagnostic features reflecting their electronic environments. A general downfield shift is observed in the signals of complex 2, consistent with the increased electron-withdrawing character of the protonated metal center, which induces deshielding of the ligand protons.

Both complexes exhibit small coupling constants between protons H_5_’ (equatorial) and H_6_ (axial), with values of 2.9 Hz and 3.0 Hz, respectively. These weak couplings suggest the formation of a single stereoisomer. They are consistent with an exo configuration of the N-acetylhistamine ligand at the C5 position of the cyclohexadienyl ring, as commonly observed in related iron tricarbonyl systems [25]. In complex 3, the olefinic protons of the cyclohexadienyl moiety (H_1_, H_4,_ and H_2_, H_3_) appear as four well-defined triplets, with coupling constants of 4.8 Hz and 4.6 Hz. This pattern indicates that the coordination of the N-acetylhistamine ligand does not significantly perturb the π-electronic structure of the organometallic fragment. The retention of signal symmetry and modest coupling values supports the delocalized nature of the cyclohexadienyl system upon ligand coordination [26].

### 3.2. Crystallographic study

**Figure 2:**
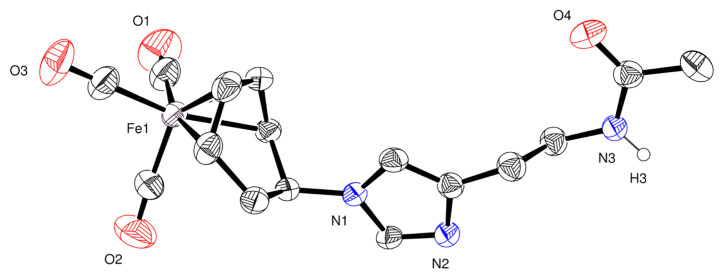
ORTEP representation of the molecular structure of complex 3, with thermal ellipsoids shown at the 50% probability level. Only heteroatoms are labeled for clarity. Hydrogen atoms are omitted, except for the amide proton.

A detailed crystallographic analysis of complex 3 is presented in Table 1, including data collection and refinement parameters. Selected bond lengths and bond angles are compiled in Tables 2 and 3, respectively. A perspective view of the molecular structure of complex 3, highlighting the coordination environment and atom labeling, is depicted in Figure 2.

**Figure 3:**
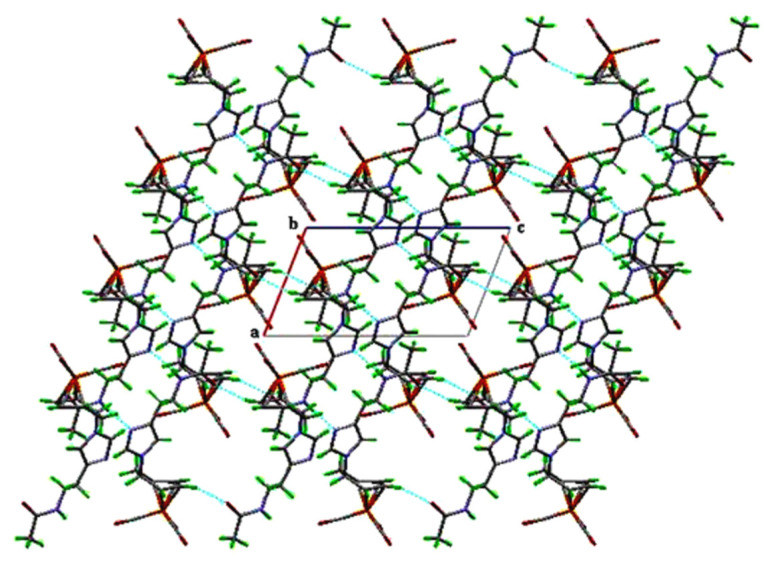
Molecular packing of complex 3 viewed along the (a, c) plane, highlighting the formation of infinite dimeric chains along the *a*-axis. Dashed lines indicate intermolecular hydrogen bonding of the N–H···N and C–H···O types.

SCXRD analysis of complex 3 confirms an exo configuration, where the N-acetylhistamine ligand is covalently bound to the cyclohexadienyl ring at C(5) via a C–N bond measuring 1.477(4) Å. The iron center adopts a distorted octahedral geometry, coordinated by three carbonyl ligands and the η^4^-cyclohexa-1,3-diene π-system. The Fe atom is slightly displaced out of the diene plane, at 0.861 and 1.627 Å from two defined planes, indicative of steric influence from the coordinated ligand. The cyclohexadienyl fragment exhibits significant puckering, with a dihedral angle of 39.27° between planes C(1)–C(2)–C(3)–C(4) and C(1)–C(6)–C(5)–C(4), larger than in unsubstituted analogues [27,28]. The Fe(CO)_3_ unit lacks ideal C_3v_ symmetry, showing two C–Fe–C angles near 100°, while the third angle (cis to the C(2)–C(3) bond) is 92.83(17)°, reflecting perturbation caused by the N-acetylhistamine ligand. The Fe–C(11) bond is slightly shorter at 1.757(4) Å compared to the average Fe–C bonds (1.785 Å), consistent with enhanced metal-to-CO back-donation facilitated by the ligand’s electron-donating character [29]. Fe–C–O angles range from 176.1(4)° to 178.5(4)°, showing minor bending attributed to intermolecular hydrogen bonding. Complex 3 forms three hydrogen bonds (N–H···N and C–H···O) (Table 4) that stabilize a three-dimensional hydrogen-bonded network. The molecular packing consists of parallel dimeric layers along the *a*-axis, as projected on the (0 ī 1) plane (Figure 3), comparable to other biologically functionalized Fe tricarbonyl complexes [30].

These structural insights demonstrate that N-acetylhistamine coordination preserves the core organometallic architecture while introducing subtle geometric and electronic modifications, highlighting its potential in the design of biorelevant Fe(CO)_3_ complexes.

### 3.3. Acid–base characterization of complex 3 [Fe(CO)_3_(1,4-η^5^-N-acetyl histaminocyclohexa-1,3-diene)]

Metal carbonyl complexes bearing functionalized organic ligands such as CO_2_H, OH, SH, or NH_2_ groups serve as sensitive molecular probes for monitoring pH-dependent behavior. Protonation or deprotonation of such ligands directly affects the carbonyl stretching vibrations, observable in the FTIR spectral window of 1800–2100 cm^−1^. In particular, the symmetric and asymmetric stretching modes of the CO ligands (υ_sym(CO) and υ_asym(CO)) exhibit distinct frequency shifts, enabling direct spectroscopic correlation with pH variations in solution [31–36]. Recent advancements in FTIR instrumentation have significantly improved both sensitivity and resolution, facilitating the simultaneous analysis of multiple carbonyl-containing complexes within mixed aqueous systems [33–35].

**Figure 4:**
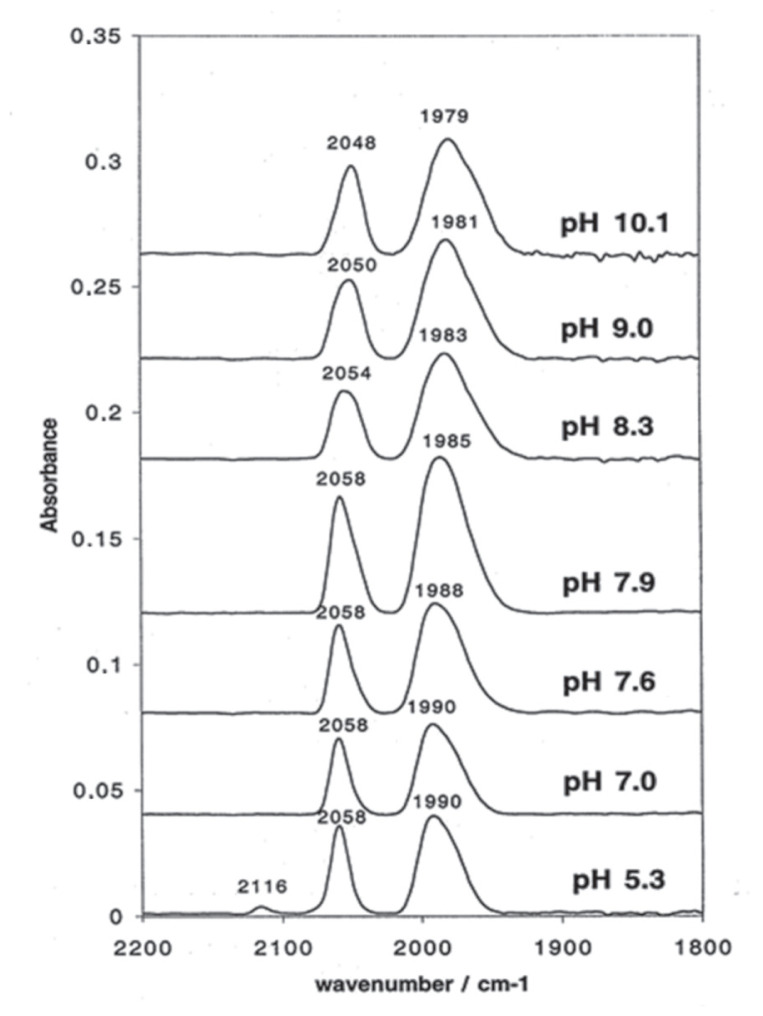
Acid–base titration of complex 3 monitored by infrared spectroscopy.

In this study, the acid–base behavior of complex 3 was monitored by FTIR spectroscopy (Figure 4) over a pH range of 5–10, using the procedure described in Section 2.3.

At pH 5.3, two ν_C≡O bands were observed at 2058 and 1990 cm^−1^, corresponding to the protonated form of the complex. A minor band at 2116 cm^−1^, attributable to the [Fe(CO)_3_(η^5^-C_6_H_7_)]^+^ cation, indicated partial dissociation of the N-acetylhistamine ligand under mildly acidic conditions. As the pH increased, the ν_C≡O bands gradually shifted to lower wavenumbers (2048 and 1979 cm^−1^), accompanied by the complete disappearance of the 2116 cm^−1^ band.

The resulting IR spectra showed clear shifts in the carbonyl stretching frequencies as a function of pH (Scheme), highlighting the spectroscopic sensitivity of the complex to its protonation state. These findings demonstrate the potential of such functionalized iron tricarbonyl complexes as pH-responsive probes for analytical applications in organometallic chemistry.

The apparent pK_a_ of the complex was estimated to be approximately 8.2 from the IR spectral transition midpoint. This value is higher than the pK_a_ of free N-acetylhistamine (≈ 6.9), suggesting that Fe(CO)_3_ coordination increases the basicity of the ligand, consistent with the increased electron density at the metal center and stabilization through π-backbonding, a phenomenon frequently observed in iron tricarbonyl complexes with nitrogen-based ligands [37–39].

Overall, complex 3 demonstrates good aqueous stability above pH 5 and remains intact up to slightly basic conditions. The increased basicity and electronic adaptability make this complex a promising scaffold for pH-responsive applications in bioorganometallic chemistry, including pH-triggered delivery systems and sensing platforms [40–41].

Although no direct IR data were recorded outside the studied pH range, the known lability of Fe(CO)_3_ complexes under extreme pH conditions suggests that the complex may undergo protonation-induced dissociation at low pH and hydrolytic degradation at high pH, potentially releasing the N-acetylhistamine ligand and generating iron carbonyl fragments such as Fe_2_(CO)_9_ or Fe_3_(CO)_12_. This hypothesis is supported by well-documented base-mediated degradation mechanisms for iron carbonyl complexes via Hieber-type reactions and hydroxide-induced CO attack [42].

## 4. Conclusion

The synthesis and characterization of the complex 3 [Fe(CO)_3_(1,4-η^5^-N-acetyl histaminocyclohexa-1,3-diene)] demonstrate the feasibility of selective organometallic tagging on a histidine-analog ligand. Structural analysis confirms a well-defined exo geometry suitable for directed functionalization. Spectroscopic data and acid–base titration reveal stability in aqueous solution and a slight increase in basicity compared to free N-acetylhistamine. These findings suggest that Fe(CO)_3_ units can serve as robust platforms for selective biomolecule labeling, paving the way for applications in bioorganometallic chemistry, particularly for recognition or modification of histidine-containing proteins.

Future developments may focus on enzymatic targeting and the design of IR-active probes for biochemical imaging.

## Supplementary Information



## Figures and Tables

**Scheme:**
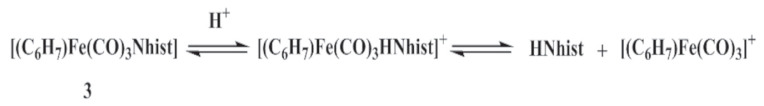
Acid–base titration profile of complex 3 bearing the N-acetylhistamine ligand (HNhist), illustrating its pH-responsive behavior in solution.

**Table 1:** Crystallographic parameters for complex 3.

Empirical formula	C_16_ H_17_ Fe N_3_ O_4_
Crystal system	Triclinic
Space group	P1̄
a (Å)	7.318(5)
b (Å)	10.611(5)
c (Å)	12.402(5)
α (°)	103.947(5)
β (°)	106.834(5)
γ (°)	100.363(5)
V (A^3^)	861.7(8)
Z	2
d_x_	1.431
F (000)	384
Reflection read	18722
Unique reflections (R _int_)	2913 (0.0463)
Data/restraints/parameters	2913/0/217
Largest diff. peak / hole (eÅ^−3^)	+0.309/−0.317
wR_2_	0.1204
R_1_ (all data)	0.0553
R_1_ (observed data)	0.0389
Goof	1.0930

**Table 2:** Selected interatomic distances (Å) in the structure of complex **3**.

Fe(1)–C(11) 1.757(4)	N(2)–C(9) 1.378(4)
Fe(1)–C(12) 1.780(4)	N(3)–C(15) 1.338(4)
Fe(1)–C(10) 1.791(4)	N(3)–C(14) 1.447(4)
Fe(1)–C(2) 2.040(4)	C(4)–C(3) 1.418(4)
Fe(1)–C(3) 2.044(3)	C(4)–C(5) 1.510(4)
Fe(1)–C(1) 2.097(3)	C(9)–C(8) 1.349(4)
Fe(1)–C(4) 2.103(3)	C(9)–C(13) 1.489(4)
N(1)–C(7) 1.343(4)	C(15)–C(16) 1.503(5)
N(1)–C(8) 1.368(4)	O(1)–C(10) 1.138(5)
N(1)–C(5) 1.477(4)	O(2)–C(11) 1.148(4)
N(2)–C(7) 1.317(4)	O(3)–C(12) 1.150(4)
C(1)–C(2) 1.410(5)	C(14)–C(13) 1.507(4)
C(1)–C(6) 1.501(4)	O(4)–C(15) 1.225(4)
C(2)–C(3) 1.394(5)	C(6)–C(5) 1.532(4)

**Table 3:** Selected bond angles (°) in the structure of complex 3.

C(11)–Fe(1)–C(12) 100.41(18)	C(11)–Fe(1)–C(10) 101.85(19)
C(12)–Fe(1)–C(10) 92.83(17)	C(11)–Fe(1)–C(2) 130.95(17)
C(12)–Fe(1)–C(2) 91.24(16)	C(10)–Fe(1)–C(2) 125.16(17)
C(11)–Fe(1)–C(3) 134.76(16)	C(12)–Fe(1)–C(3) 120.77(16)
C(10)–Fe(1)–C(3) 94.48(17)	C(2)–Fe(1)–C(3) 39.93(14)
C(11)–Fe(1)–C(1) 91.70(16)	C(12)–Fe(1)–C(1) 93.09(15)
C(10)–Fe(1)–C(1) 163.98(16)	C(2)–Fe(1)–C(1) 39.82(13)
C(3)–Fe(1)–C(1) 69.74(14)	C(11)–Fe(1)–C(4) 96.70(15)
C(12)–Fe(1)–C(4) 160.31(16)	C(10)–Fe(1)–C(4) 93.14(15)
C(2)–Fe(1)–C(4) 70.05(13)	C(3)–Fe(1)–C(4) 39.98(12)
C(1)–Fe(1)–C(4) 76.65(13)	C(7)–N(1)–C(8) 105.8(3)
C(7)–N(1)–C(5) 126.2(3)	C(8)–N(1)–C(5) 127.4(3)
C(7)–N(2)–C(9) 104.8(2)	C(15)–N(3)–C(14) 122.2(3)
C(3)–C(4)–C(5) 120.2(3)	C(3)–C(4)–Fe(1) 67.78(18)
C(5)–C(4)–Fe(1) 108.31(19)	C(8)–C(9)–N(2) 109.3(3)
C(8)–C(9)–C(13) 129.5(3)	N(2)–C(9)–C(13) 121.1(3)
C(9)–C(8)–N(1) 107.4(3)	C(2)–C(1)–C(6) 119.9(3)
C(2)–C(1)–Fe(1) 67.9(2)	C(6)–C(1)–Fe(1) 109.8(2)
C(3)–C(2)–C(1) 115.2(3)	C(3)–C(2)–Fe(1) 70.19(19)
C(1)–C(2)–Fe(1) 72.3(2)	O(4)–C(15)–N(3) 121.6(3)
O(4)–C(15)–C(16) 122.6(3)	N(3)–C(15)–C(16) 115.8(3)
C(2)–C(3)–C(4) 115.4(3)	C(2)–C(3)–Fe(1) 69.9(2)
C(4)–C(3)–Fe(1) 72.25(19)	C(1)–C(6)–C(5) 110.7(3)
O(2)–C(11)–Fe(1) 176.1(4)	O(3)–C(12)–Fe(1) 177.7(4)
O(1)–C(10)–Fe(1) 178.5(4)	N(1)–C(5)–C(4) 111.4(2)
N(1)–C(5)–C(6) 111.2(3)	C(4)–C(5)–C(6) 110.9(2)
N(3)–C(14)–C(13) 112.4(3)	C(9)–C(13)–C(14) 113.9(3)
N(2)–C(7)–N(1) 112.7(3)	

**Table 4:** Intermolecular hydrogen-bonding interactions observed in complex 3.

D–H…A	D–H (Å)	H...A (Å)	D…A (Å)	D–H…A (°)
N(3)–H(2A)…..N(2)^i^	0.859	2.128	2.985	174.31
C(4)–H(4)….O(1)^ii^	0.979	2.618	3.511	151.67
C(3)–H(3)….O(1)iii	0.980	2.507	3.164	124.29

Symmetry codes:

i (− 1− x, − y, 1 − z);

ii (1 + x, y, z);

iii (− x, 1 − y, 2 − z)
